# How and When to Drink Water

**Published:** 1890-02

**Authors:** 


### HOW AND WHEN TO DRINK WATER.

According to Dr. Leuf, when water is taken into the full or partly-full stomach, it does not mingle with the food, as we are taught, but passes along quickly between the food and lesser curvative toward the pylorus, through which it passes into the intestines. The secretion of mucus by the lining membrane is constant, and during the night a considerable amount accumulates in the stomach; some of its liquid portion is absorbed, and that which remains is thick and tenacious. If food is taken into the stomach when in this condition it becomes coated with this mucous, and the secretion of the gastric juice and its action are delayed. These facts show the value of a goblet of water before breakfast. This washes out the tenacious mucous, and stimulates the gastric glands to secretion. In old and feeble persons water should not be taken cold, but it may be with great advantage taken warm or hot. This removal of the accumulated mucous from the stomach is probably one of the reasons why taking soup at the beginning of a meal has been found so beneficial.

